# Living on the Edge of the CNS: Meninges Cell Diversity in Health and Disease

**DOI:** 10.3389/fncel.2021.703944

**Published:** 2021-07-01

**Authors:** Julia Derk, Hannah E. Jones, Christina Como, Bradley Pawlikowski, Julie A. Siegenthaler

**Affiliations:** ^1^Section of Developmental Biology, Department of Pediatrics, University of Colorado, Aurora, CO, United States; ^2^Cell Biology, Stem Cells and Development Graduate Program, University of Colorado, Anschutz Medical Campus, Aurora, CO, United States; ^3^Neuroscience Graduate Program, University of Colorado, Aurora, CO, United States

**Keywords:** meninges, fibroblast, meningeal lymphatic system, arachnoid barrier, blood-CSF barrier, border-associated macrophages

## Abstract

The meninges are the fibrous covering of the central nervous system (CNS) which contain vastly heterogeneous cell types within its three layers (dura, arachnoid, and pia). The dural compartment of the meninges, closest to the skull, is predominantly composed of fibroblasts, but also includes fenestrated blood vasculature, an elaborate lymphatic system, as well as immune cells which are distinct from the CNS. Segregating the outer and inner meningeal compartments is the epithelial-like arachnoid barrier cells, connected by tight and adherens junctions, which regulate the movement of pathogens, molecules, and cells into and out of the cerebral spinal fluid (CSF) and brain parenchyma. Most proximate to the brain is the collagen and basement membrane-rich pia matter that abuts the glial limitans and has recently be shown to have regional heterogeneity within the developing mouse brain. While the meninges were historically seen as a purely structural support for the CNS and protection from trauma, the emerging view of the meninges is as an essential interface between the CNS and the periphery, critical to brain development, required for brain homeostasis, and involved in a variety of diseases. In this review, we will summarize what is known regarding the development, specification, and maturation of the meninges during homeostatic conditions and discuss the rapidly emerging evidence that specific meningeal cell compartments play differential and important roles in the pathophysiology of a myriad of diseases including: multiple sclerosis, dementia, stroke, viral/bacterial meningitis, traumatic brain injury, and cancer. We will conclude with a list of major questions and mechanisms that remain unknown, the study of which represent new, future directions for the field of meninges biology.

## Introduction

The meninges are the multifaceted structure surrounding the brain and spinal cord with three structurally and cellularly distinct layers: the pia, arachnoid, and dura. The meninges house a variety of cell types including the largest population of CNS fibroblasts, three different vascular networks, specialized immune populations, neural stem cells and suture stem cells. The meninges were once considered a simple protective structure, but we now understand that the diversity of cells found in the meninges mediate multiple CNS functions. In this review, we will detail the different cell populations of the meninges and discuss their role in CNS development, homeostasis, injury, and disease.

## Meningeal Cell Types

**FIGURE 1:**
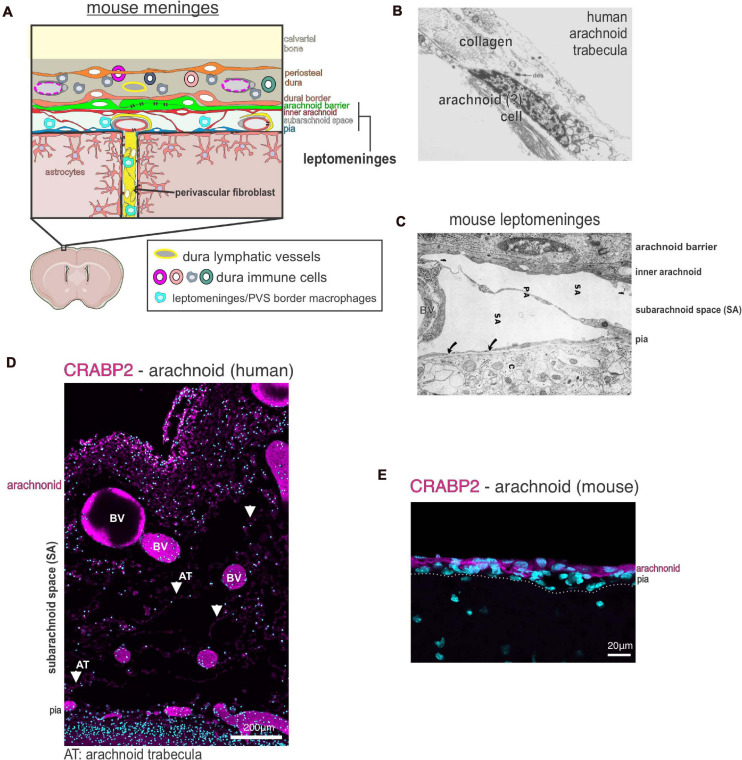
Meninges structure and cellular heterogeneity **(A)** Schematic of cellular make up and structure of the mouse meninges and contiguous perivascular space. **(B)** Electron microscopy image of a human arachnoid trabecula, the collagen fibril structures that span the wide of the sub-arachnoid space. A cell, potentially an arachnoid, is seen associated with the collagen fibril. Image reproduced with permission from Alcolado et al. (1988). **(C)** Electron microscopy image of the mouse leptomeninges, a “pia-arachnoid” cell process (PA) spans the subarachnoid space (SA) containing blood vessels (BV). Cells of the inner arachnoid are immediately adjacent to the arachnoid barrier cell layer, which contains microfibrils (f) of extracellular matrix material. Image reproduced with permission from McLone and Bondareff (1975). **(D)** Immunofluorescence image of human fetal leptomeninges in the Sylvian sulcus labeled with CRABP2 (magenta) and DAPI (cyan). CRABP2 immunoreactivity in the meninges is limited to the arachnoid layer and cells associated with the arachnoid trabecula (AT) in the subarachnoid space (SA). Image reproduced with permission from DeSisto et al. (2020). BV, blood vessels. **(E)** Immunofluorescence image of mouse leptomeninges at postnatal day 14 labeled with CRABP2 (magenta) and DAPI (cyan). CRABP2 immunoreactivity is seen in cells of the arachnoid layer but not in pia-located cells. Image reproduced with permission from DeSisto et al. (2020).

The meninges contain two compartments: the leptomeninges (collective term for pia and arachnoid layers) and the dura (Figure 1A). The vascular make-up, fibroblast, and immune cell populations are different between the two compartments, as are their roles in development, homeostasis, and disease. In this section, we will review the cellular, molecular, and developmental identities of meningeal cells, with a particular focus on the fibroblast populations.

### Fibroblasts

Fibroblasts, once thought of as purely structural cells, are now known to execute a variety of functions throughout the body. These functions, which are often organ and tissue specific, include the regulation of neighboring blood vessels, immune cells, and lymphatic vessels, through the production of growth factors, cytokines, and extracellular matrix remolding (Liu et al., 2008; Mueller and Germain, 2009; Barron et al., 2016; Chapman et al., 2016; Furtado, 2016; Roulis and Flavell, 2016; Pikor et al., 2017; Buechler and Turley, 2018; Tallquist, 2020; Wang et al., 2020). Consistent with diverse roles, single cell transcriptome studies of the CNS and non-CNS organs demonstrate transcriptional heterogeneity in fibroblast populations that can correlate with specific locations within a tissue (Saunders et al., 2018; Vanlandewijck et al., 2018; Dobie et al., 2019; Tsukui et al., 2020). Our recent single cell RNA sequencing (scRNAseq) of developing mouse meninges showed pia, arachnoid and dura fibroblasts are molecularly distinct and likely have layer specific functions (DeSisto et al., 2020). However, we are just beginning to understand how the molecular and cellular features of meningeal fibroblasts emerge during development and how these specializations translate to function.

Primitive, non-layer specific meningeal fibroblasts are first observed as a mesenchymal layer called the “primary meninx” which surrounds the nervous system by embryonic day 10.5 (E10.5) in the mouse (McLone and Bondareff, 1975; Dasgupta and Jeong, 2019). Meningeal fibroblasts in different CNS regions have different origins; forebrain meningeal fibroblasts are neural crest derived whereas mid-, hindbrain and spinal cord meninges are derived from the mesoderm (Jiang et al., 2002; Yoshida et al., 2008). By E12, the mouse meninges is organized into a thin outer layer of flat cells and a loose inner layer, which show distinct molecular profiles (Dasgupta et al., 2019; DeSisto et al., 2020). By E13.5 the three layers of meninges (pia, arachnoid, and dura) can be identified in the ventral forebrain, with fibroblasts from each layer having established unique transcriptional signatures and cellular specializations (DeSisto et al., 2020). Maturation and differentiation of the meningeal layers continues in a ventral to dorsal pattern over the forebrain (Vivatbutsiri et al., 2008; Siegenthaler et al., 2009; DeSisto et al., 2020).

The pia is a single layer of fibroblasts that sits adjacent to the glia limitans, a basement membrane (BM) that contacts the brain parenchyma and serves as the attachment point for radial glia cells during development and later for astrocytic end feet (McLone and Bondareff, 1975; Zhang et al., 1990; Sievers et al., 1994; Beggs et al., 2003). The transcriptional profile of pial fibroblasts shows enriched expression of extracellular matrix (ECM) genes, many of which are key components of the pial basement membrane (DeSisto et al., 2020). This supports pial fibroblasts as critical for pial BM maintenance. Embryonic pial cells uniquely express genes not seen in arachnoid or dura fibroblasts, such as *S100a6* and *Ngfr*, and have sub-populations that correlate with specific brain regions (DeSisto et al., 2020). The functional relevance of pial fibroblast heterogeneity in the developing meninges is not known nor is it known if this persists in adult meninges.

Arachnoid fibroblasts are organized into a meshwork of column-like trabecular structures that create space for large blood vessels and open pockets for cerebrospinal fluid (CSF) (Figure 1). Studies using transmission and scanning EM in rat and human show arachnoid trabeculae are pillars of collagen fibrils surrounded by fibroblasts (Alcolado et al., 1988; Saboori and Sadegh, 2015; Mortazavi et al., 2018; Figure 1B). Trabecular pillars are not seen in mouse arachnoid cells, and EM studies instead show long cellular processes of fibroblasts that span the subarachnoid space (McLone and Bondareff, 1975; Figure 1C). Fibroblasts associated with human arachnoid trabeculae have been previously described in EM studies as un-specialized leptomeningeal fibroblasts (Alcolado et al., 1988). However, we found that the molecular profile of human and mouse arachnoid fibroblasts, including in human those associated with trabecula, differs from other leptomeningeal fibroblasts in the pia. Arachnoid fibroblasts express RALDH2 and CRABP2, a retinoic acid (RA) synthesizing enzyme and an RA binding protein, respectively (Figures 1D,E; DeSisto et al., 2020). Production of RA by the arachnoid layers critically regulates brain and neurovascular development (Siegenthaler et al., 2009; Choi et al., 2014; Bonney et al., 2016; Mishra et al., 2016; Haushalter et al., 2017). Arachnoid fibroblasts are also uniquely enriched in other secreted factors such as *Wnt6*, *Angptl2*, and *Bmp4* that may act locally in other meninges cells or adjacent structures such as the brain or calvarium (Dasgupta et al., 2019; DeSisto et al., 2020).

Fibroblasts in the mouse dura are organized into an outer layer that adhere to the underside of calvarium bones and an inner layer that contact arachnoid barrier cells (Figure 1A). Fibroblasts in the outer dural layer are essentially the periosteal cells of the calvarial bones and consist of abundant collagen fibrils. The inner layer of dural fibroblasts are called dural border cells and are immediately adjacent to arachnoid barrier cells (Nabeshima et al., 1975; Alcolado et al., 1988). Of note, a scRNAseq study of the developing mouse cranial suture indicate that outer periosteal dural fibroblasts and inner dural border fibroblasts are likely molecularly distinct fibroblast populations (Farmer et al., 2021). Additionally, dural fibroblasts have been shown to play a role in suture patency during calvarium expansion to accommodate brain growth (Cooper et al., 2012; Yu et al., 2021).

The last class of fibroblasts seen in the meninges are perivascular fibroblasts (Figure 1A). Perivascular fibroblasts are found around large diameter blood vessels throughout the pia and sit immediately adjacent to the vascular smooth muscle layer (Zhang et al., 1990; Hannocks et al., 2018; Riew et al., 2020; Bonney et al., 2021; Sato et al., 2021). Perivascular fibroblasts are also found around penetrating arterioles and pre-capillary arterioles but not capillaries in the CNS parenchyma of human (Zhang et al., 1990) and rodent (Soderblom et al., 2013; Kelly et al., 2016; Hannocks et al., 2018; Bonney et al., 2021; Dorrier et al., 2021). Recent work using 2-photon live imaging in adult mice detailed the topography of perivascular fibroblasts, showing that perivascular fibroblasts in the brain parenchyma can extend over 200μm on cerebral penetrating arterioles but only extend very short distances on ascending venules (Bonney et al., 2021). Studies of human and rodent meninges described perivascular fibroblasts as “pial” or “leptomeningeal” cells that form an adventitial layer (Zhang et al., 1990; Hannocks et al., 2018; Riew et al., 2020). However, the exact molecular identity of perivascular fibroblasts has not yet been fully elucidated. For example, are perivascular fibroblasts a homogenous population or are the specialized based on tissue location (meninges vs. parenchyma) or type of blood vessel they surround? It is also unknown how perivascular fibroblasts compare to non-vascular meningeal fibroblasts (such as those in the arachnoid or arachnoid barrier). Do they differ transcriptionally, developmentally, or functionally? Perivascular fibroblasts share some of the same markers as pial fibroblasts such as collagen-1, laminin α1 and Platelet-derived growth factor receptor-α (PDGFRα) (Kelly et al., 2016; Hannocks et al., 2018; Vanlandewijck et al., 2018). Perivascular fibroblasts are of high interest in the context of brain injury and neurological disease because their activation contributes to fibrotic scar formation and inflammation (discussed in detail below). Very little is known about perivascular fibroblasts during CNS development or adult homeostasis, and we expect that future work detailing perivascular fibroblasts will yield important results.

### Arachnoid Barrier Cells

**FIGURE 2:**
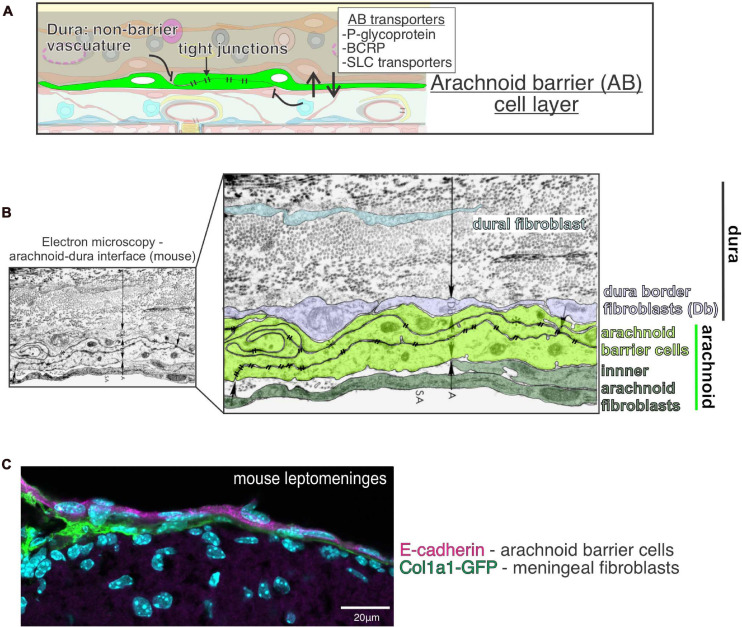
Function and structure of the arachnoid barrier layer in the meninges. **(A)** Graphical depiction of proposed functions of the arachnoid barrier layer of the meninges including (1) as a physical barrier preventing free movement of molecules and cells into the subarachnoid space by virtue of its tight junctions, (2) enriched expression of efflux (P-glycoprotein, BCRP) and solute transporters (SLC) by arachnoid barrier cells support regulated movement of molecules across this barrier layer. **(B)** Electron microscopy image of the mouse arachnoid-dura interface, reproduced with permission from Nabeshima et al. (1975). Pseudo-coloring of the cell bodies highlights close interface between cells of the arachnoid (A) (inner arachnoid fibroblasts and arachnoid barrier cells connected by electron-dense tight junctions) and cells of the dura, [dura border cells (Db) and dural fibroblasts within collagen fibril dense dura layer]. **(C)** Immunofluorescence image of mouse leptomeninges from a postnatal day 14 *Col1a1-GFP* mouse brain with E-cadherin (magenta) and DAPI (cyan). E-cadherin is expressed by arachnoid barrier cells (representing the outer part of the arachnoid layer) but not by inner *Col1a1-GFP+* fibroblasts, representing pial and inner arachnoid fibroblasts. Image reproduced with permission from DeSisto et al. (2020).

The arachnoid barrier, part of the blood CSF-barrier, is an epithelial-like cell layer that separates and controls transport between the dura and CSF filled subarachnoid space (Saunders et al., 2008, 2013; Figures 1, 2). The subarachnoid space, defined by the arachnoid barrier cells on top and pia below, is continuous with perivascular spaces in the brain parenchyma, meaning that molecules in the CSF can access brain tissue (Iliff et al., 2012; Hannocks et al., 2018). Thus, the arachnoid barrier creates a border between the fenestrated vasculature of the dura and CNS (Figure 2A). Arachnoid barrier cells express a variety of transporters and can be altered by disease or infection (detailed below), but few functional studies have been done on the arachnoid barrier in comparison to other CNS barriers like the blood brain barrier (BBB). Further, little is known regarding arachnoid barrier development. We recently found that arachnoid barrier cells can be first seen in the mouse forebrain around E13 and they differentiate from mesenchymal cells of the primary meninx (DeSisto et al., 2020).

EM studies first illuminated that the arachnoid barrier cells are laden with tight junctions over 40 years ago (Nabeshima et al., 1975; Figure 2B). These findings were preceded by tracer studies demonstrating a functional barrier between the dura and subarachnoid space (Rodriguez, 1955), later confirmed using horseradish peroxidase (HRP) tracer (Balin et al., 1986). Tight junction containing arachnoid barrier cells are observed in the meniges of multiple species from jawless fish to humans, demonstrating evolutionary conservation (Nabeshima et al., 1975; Nakao et al., 1988; Vandenabeele et al., 1996; Rascher and Wolburg, 1997; Brøchner et al., 2015). Arachnoid barrier cells express the tight junction protein Claudin 11, best known for its role in the blood-testes barrier and maintenance of myelin wraps, along with E-Cadherin, consistent with their epithelial-like identity (Figure 2C; Rascher and Wolburg, 1997; Bhatt et al., 2013; Brøchner et al., 2015; Uchida et al., 2019b; Ximerakis et al., 2019; DeSisto et al., 2020).

Like other CNS barrier cells, arachnoid barrier cells are enriched in a variety of transporters, including: ABCB1 (Pglycoprotein or Pgp), ABCG2 (BCRP), ABCC4 (MRP4), and SLC transporters OAT1 (Slc22a6) and OAT3 (Slc22a8) (Figure 2A; Ek et al., 2010; Møllgård et al., 2017; Yaguchi et al., 2019). Functional studies show that arachnoid barrier OAT1 and OAT3 participate in solute clearance out of the CSF (Yasuda et al., 2013; Uchida et al., 2019a). The exact role for arachnoid barrier cells in controlling CSF composition and CNS transport is still unclear, but it’s likely that a detailed understanding of arachnoid barrier cell function could be leveraged to improve CNS drug delivery. Finally, understanding if the arachnoid barrier plays a role in maintaining the vascular and immune specialization seen in the dura and leptomeninges (detailed below) could hold clinical importance.

### Blood Vessels: Leptomeninges and Dura

The blood vessels of the leptomeninges, often referred to as the pial vasculature, are connected to the parenchymal CNS vasculature, and located in the subarachnoid space. Several studies show this vasculature has barrier properties and are therefore part of the meninges blood-CSF barrier. Structural studies show that pial blood vessels have tight junctions, including expression of occludin and claudin proteins, as well as adherens junctions, that link to actin filaments (Nabeshima et al., 1975; Nakao, 1979; Cassella et al., 1997; Mazaud-Guittot et al., 2010; Zihni et al., 2016; Liebner et al., 2018; Rua and McGavern, 2018). Further, the pial vasculature is not permeable to peripheral injections of horseradish peroxidase (44 kD) (Balin et al., 1986), and has a high trans-endothelial electrical resistance (Butt et al., 1990; Revest et al., 1994). Pial blood vessels lack expression of PLVAP (Daneman et al., 2010), a component of blood vessels fenestrations, and show high expression of glucose transporter GLUT-1 (Sabbagh et al., 2018). Of note, the pial vascular plexus lacks proximate astrocytic end feet, which the BBB possesses pervasively (Blanchette and Daneman, 2015). Pial vasculature also lacks capillaries; thus, the relevance of its barrier properties may serve more in controlling the movement of immune cells and other blood contents into and out of CSF than fine-tuning solute transport. Overall, the barrier structures seen in the pial vasculature regulates the free movement of molecules and cells into the CSF and CNS.

The pial vasculature forms via vasculogenesis starting at E8 in the mouse, first around the spinal cord and expanding to encapsulate the forebrain starting at E10. This developmental blood vessel system is referred to as the perineural vascular plexus (PNVP) (Nakao et al., 1988). PNVP formation is initiated by VEGFA secretion from the neural tube (Hogan et al., 2004) and is strongly influenced by fibroblasts of the meninges. For example, retinoic acid produced by arachnoid fibroblasts regulates endothelial Wnt-ß-catenin signaling to promote PNVP growth (Mishra et al., 2016). Endothelial Wnt-ß-catenin signaling likely controls the acquisition of barrier properties in the PNVP, as it does in parenchymal vasculature (Zhou et al., 2014; Mishra et al., 2016). Interestingly, VEGF and Wnt ligands are expressed by pia and arachnoid fibroblasts, respectively, during development (DeSisto et al., 2020) and these signals likely regulate PNVP development. The PNVP continues to grow and mature postnatally (Coelho-Santos and Shih, 2020), but the exact role for the meningeal fibroblasts at these later time points is currently unknown.

The dura contains an extensive network of blood vessels that include arteries, veins, and fenestrated capillary beds (Shukla et al., 2002, 2003; Coles, 2017; Mecheri et al., 2018). A unique feature of the dura vasculature is the presence of multiple large veins called dural venous sinuses which serve as the main exit for blood from the brain via the cerebral veins (Shukla et al., 2003; Coles, 2017; Mecheri et al., 2018). The non-barrier, fenestrated blood vessels of the dura, are permeable to horseradish peroxidase (MW 44 kD) administered intravenously (Balin et al., 1986) and allow non-selective movement of cells and molecules from the peripheral circulatory system into the dura. The proximity of the leptomeningeal barrier vasculature and non-barrier vasculature of the dura, especially in animals with thin meninges like rodents, raises the question, what are the mechanisms that maintain these specialized properties? Embryonic dural fibroblasts are enriched in Wnt inhibitors *Dkk2* and *Sfrp1* which could prevent development of barrier properties in the dural blood vasculature as occurs in other circumventricular organs (Benz et al., 2019; Wang Y. et al., 2019; DeSisto et al., 2020). Many details of dural blood vessel plexus development have yet to be elucidated. Development of the dura venous sinuses starts around E12 and occurs through the remodeling of three developmental venous plexuses (Tischfield et al., 2017). Proper development of dura venous sinus veins requires paracrine BMP signaling from skull progenitors cells and dural fibroblasts and is disrupted in skull malformations such as craniosynostosis (Tischfield et al., 2017). Away from the dural sinuses, recent work showed that between P0 to P28 there is a gradual reduction in dural blood vessel density and branching (Sato et al., 2021) however, the molecular pathways controlling initial dura blood vascular plexus growth and refinement have so far not been studied in any detail.

### Lymphatic Vessels: Dura

Although the structure of lymphatic vessels was first described in 1787 by Mascagni, scientific literature consistently purported that the CNS completely lacked lymphatic vasculature until these vessels were “rediscovered” in 2015 (Bucchieri et al., 2015; Louveau et al., 2015). The dural lymphatics are now recognized as a critical transport system of macromolecules, interstitial fluid, and CSF from the CNS into the cervical lymph nodes (Aspelund et al., 2015; Absinta et al., 2017; Louveau et al., 2018; Ahn et al., 2019). Further, the altered function of dural lymphatics is now implicated in several neurodegenerative diseases (Louveau et al., 2017; Da Mesquita et al., 2018b, 2021).

The lymphatic system in the dura does not form until after birth in the mouse, much later than the peripheral lymphatic system (Izen et al., 2018) and meningeal blood vessels (reviewed above). Dural lymphatic vessels are composed of specialized endothelial cells that express VEGFR-3, LYVE-1, SLC, PDPN, and Prox1 and require VEGF-C signaling and lymphatic flow to properly mature (Antila et al., 2017; Izen et al., 2018; Bálint et al., 2019). By approximately postnatal day 20 in a mouse, the intracranial lymphatic vessels that line the dural sinuses and the extracranial lymphatic vessels that abut cranial nerves, become fully functional, draining content from the CNS into the peripheral lymphatic system at the base of the skull (Aspelund et al., 2015; Antila et al., 2017; Izen et al., 2018; Rustenhoven et al., 2021). Interesting, the dural lymphatic vasculature network is more voluminous in the ventral portions of the skull and display increased complexity, including valves, which the superior lymphatics lack (Ahn et al., 2019).

The three vascular plexuses of the meninges (pial, dural, lymphatics) are strikingly diverse in their developmental timing, barrier properties, and the functions they serve. The variations in barrier integrity have major implications in waste drainage, immune trafficking, antigen presentation, and drug delivery to the brain. Overall, the meningeal vascular systems are critical to CNS function.

### Immune Cells

The meninges house an extensive immune cell population that are increasingly recognized to execute important functions in the CNS. The immune cells present in the leptomeninges differ from those present in the dura and several recent reviews detail the transcriptional and functional diversity of meningeal immune cells (Rua and McGavern, 2018; Kierdorf et al., 2019; Alves de Lima et al., 2020). Specifically, Rua and McGavern provide important details for T cells including that CD4+ T cells traffic from the blood to the dura and then the deep cervical lymph nodes, potentially “scanning” the dura meningeal tissue before returning to the lymph node; they also summarize how CD4+ T and B cells mediate homeostatic behavior and that these cells are enriched in the dura with aging. Kierdorf et al. (2019)’s review delves into the barriers that segregate macrophages into various compartments in the CNS at the dura, leptomeninges, choroid plexus, and perivascular compartments and how these populations are differentially regulating homeostasis and disease processes. Finally, Alves de Lima et al. (2020) is a meticulous review of meningeal immune compartments with important speculation on the future directions and importance of meningeal immune cell function. Our goal with this section is to give an overview of meningeal immune cells under homeostatic conditions and direct readers to comprehensive reviews for further details, such as the ones listed above.

The adult leptomeninges primarily harbor macrophages and non-migratory dendritic cells, along with much smaller numbers of lymphoid cells (Mrdjen et al., 2018; Jordão et al., 2019; Van Hove et al., 2019). The adult dura contains macrophages, mast cells, B cells, T cells, neutrophils, innate lymphoid cells, and the largest population of dendritic cells, including migratory dendritic cells, in the CNS (Mrdjen et al., 2018; Jordão et al., 2019; Van Hove et al., 2019). Of note, immune cells of the dura are not evenly distributed through the tissue but rather accumulate around dural venous sinuses (Rustenhoven et al., 2021). Dural venous sinuses are an active site of immune cells trafficking and have emerged as a key neuroimmune interface (Rustenhoven et al., 2021). Stromal cells of the dural sinuses (mural cells and fibroblasts) promote immune cell trafficking and T cell extravasation through their expression of ICAM, VCAM, and Cxcl12 (Rustenhoven et al., 2021). T cells interact with dural antigen-presenting cells laden with CSF derived antigens (Rustenhoven et al., 2021), representing a novel mode of peripheral immune cell surveillance of the CNS. In addition to T cells, gut-educated IgA+ B cells also localize next to the dural venous sinuses to protect against bacterial and fungal brain infection (Fitzpatrick et al., 2020).

Macrophages of the meninges are one of better documented meningeal immune cell and belong to a highly specialized class of macrophages called border associated macrophages (BAMs). BAMs and microglia both originate from yolk sac erythro-myeloid progenitors and can be detected in the brain as early as E10 (Utz et al., 2020). As development continues BAMs and microglia segregate both physically and transcriptionally, with BAMs remaining in the leptomeninges (they are also in the choroid plexus and perivascular spaces) and expressing *CD206* and *Lyve1* which are not expressed by microglia. In the adult, leptomeningeal BAMs are defined by expression of *CD206*, *Lyve1*, *P2rx7*, and *Egfl7* and have significantly different transcriptional profiles from dural BAMs (Mrdjen et al., 2018; Van Hove et al., 2019). Adult dural BAMs don’t express *Lyve1* and can also be divided in subgroups. For example, one group of dural BAMs has low expression of major histocompatibility complex II (MHCII^*lo*^) and express *Clec4n*, *Clec10a*, *Folr2*, while MHCII^*hi*^ dural BAMs express greater CCR2, implicating a monocytic origin. Another important difference is that leptomeningeal BAMS are long lived while dural BAMs are continuously renewed by peripheral monocytes (Goldmann et al., 2016; Van Hove et al., 2019). The bone marrow in the calvarium and vertebral column specifically supply monocytes and neutrophils to the dura during homeostasis (Cugurra et al., 2021) and to the meninges and brain parenchyma following brain injury or in neuroinflammation via vascular tunnels connecting the bone marrow and dura (Herisson et al., 2018; Yao et al., 2018; Cai et al., 2019; Cugurra et al., 2021). The unique properties seen among leptomeningeal and dural BAMs is consistent with specialized functions for these populations in their respective barrier and non-barrier compartments.

While several studies have begun to investigate BAMs in disease (discussed below) very little has been worked out regarding their function in development or homeostasis. Further, the investigations looking into to the details and functions of the other meningeal immune cell populations have only just started.

## Meningeal Response to Injury and Disease

**FIGURE 3:**
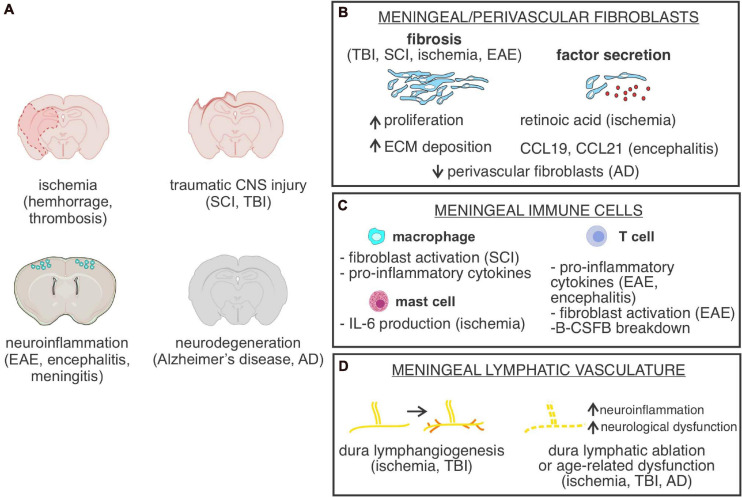
Meningeal cells or structures in CNS injury and disease. **(A)** Depiction of CNS injury or diseases in which meningeal cells, meninges located cell types or parenchyma located perivascular fibroblasts are part of the pathology. SCI, spinal cord injury; TBI, traumatic brain injury; EAE, experimental autoimmune encephalomyelitis. **(B)** Graphical depiction of fibrosis, caused by increased meningeal/perivascular fibroblast proliferation and Extracellular matrix (ECM) deposition and summary of known CNS fibroblast-derived factors identified in specific CNS disease states. AD, Alzheimer’s disease. **(C)** Summary of functional roles of meningeal-located immune cells in specific CNS disease states. B-CSFB, blood-CSF barrier. **(D)** Summary of cellular changes in meninges located lymphatic vasculature in response to different CNS injuries and disease states.

The next sections will highlight how meningeal cell types and structures respond to CNS injury (traumatic brain injury, stroke, spinal cord injury), infections (meningitis), and disease (multiple sclerosis, cancer, Alzheimer’s disease) (Figure 3). We also recommend several other comprehensive recent reviews that highlight the meningeal vasculature, immune cells, and lymphatics/glymphatics in disease (Rasmussen et al., 2018; Rua and McGavern, 2018; Mastorakos and McGavern, 2019; Alves de Lima et al., 2020; Bolte and Lukens, 2021).

### Meningeal and Perivascular Cells in Acute CNS Injury

Acute injuries to the CNS, such as stroke, traumatic brain injury (TBI) or spinal cord injury (SCI), induce a cascade of immediate events beginning with BBB breakdown, peripheral immune infiltration, acute inflammation, and tissue edema, which ultimately leads to neuronal cell loss (reviewed in Mastorakos and McGavern, 2019). These immediate events are followed by a protracted formation of fibrotic and glial scars, which have both neuroprotective and detrimental effects (Hesp et al., 2018). CNS acute injury responses are driven by a variety of CNS parenchymal cells, perivascular cells and meningeal cell types. For example, formation of the glial scar, which is largely driven by astrocytes, prevents peripheral immune cell invasion and limits inflammation by “sealing” off the infarct area from the healthy CNS tissue. Further, the glial scar works together with the fibrotic scar, which is primarily generated by meningeal and perivascular fibroblasts, to facilitate wound healing (Bundesen et al., 2003; Kelly et al., 2016; Dias and Göritz, 2018; Hesp et al., 2018; Riew et al., 2018). However, both the glial and fibrotic scars also act as major barriers to neural regeneration and axon regrowth long-term (Hesp et al., 2018). The contradictory effects of scar formation underscore the importance of investigating the cell and molecular mechanisms that drive post injury astrocyte and fibroblast behavior. Beyond astrocytes and fibroblasts, other perivascular and meningeal cells and structures like immune cells and lymphatics, also contribute to the CNS injury acute injury response and are discussed in more detail below.

### CNS Fibroblasts in Acute CNS Injury

There is ample evidence for fibrotic scar formation following acute CNS injury, but there is some confusion regarding the exact identity of the fibrotic scar forming cells. Beginning ∼3 days post injury, the fibrotic scar forming cells, which are located perivascularly and in all three layers of the meninges, become activated; characterized by proliferation, detachment from the vasculature, increased expression of ECM molecules (vimentin, fibronectin, type I collagens), and upregulation of smooth muscle actin (SMA) (Fernández-Klett et al., 2013; Soderblom et al., 2013; Kelly et al., 2016; Dias and Göritz, 2018; Riew et al., 2018; Figure 3B). The fibrotic scar forming cells express PDGFRβ [which is expressed by fibroblasts, pericytes and vascular smooth muscle cells (vSMCs)], along with PDGFRα, and Collagen-1, which are expressed by fibroblasts, but not pericytes or vSMCs (Soderblom et al., 2013; Kelly et al., 2016; Vanlandewijck et al., 2018). Fibrotic scar forming cells are labeled in the *Collagen1a1-GFP* mouse line, which marks fibroblasts but not pericytes, and do not express common markers of pericytes, like desmin and Ng2 (Göritz et al., 2011; Soderblom et al., 2013; Kelly et al., 2016). Thus, the expression pattern of fibrotic scar forming cells is consistent with that of fibroblasts, which are found in both the leptomeninges and CNS perivascular spaces (Soderblom et al., 2013; Kelly et al., 2016). Further support was demonstrated in a mouse model of multiple sclerosis, where the authors used a *Col1a2-CreER*T mouse line, which labels all CNS fibroblasts but not pericytes or vSMCs, with a Cre reporter and found that almost all fibrotic cells within the lesion were labeled by the reporter (Dorrier et al., 2021). In contrast, *Ng2-CreERT* lineage traced cells, which includes pericytes, vSMCs and some neural populations, did not show any expansion in the lesion (Dorrier et al., 2021). While not done in an acute injury model, this evidence further supports that CNS fibroblasts, not pericytes or vSMCs, are the main reactive fibrotic cell type in CNS injuries and disease. It should be noted that some of the confusion surrounding fibrotic scar forming cells comes from a lack of established nomenclature, as scar forming cells are sometimes called stromal cells, mesenchymal cells, Type A Pericytes, Type 2 pericytes and fibroblasts (Göritz et al., 2011; Dias et al., 2018, 2020). In summary, fibroblasts from the meninges and perivascular space are major drivers of fibrotic scar formation following CNS injury however establishing a standard nomenclature would be beneficial.

Another major function of CNS fibroblasts following acute CNS injury is communication with neighboring cells (Figure 3B). For example, in a rat spinal cord transection model of SCI, direct signaling between fibrotic cells and astrocytes mediated the glial/fibrotic scar border formation (Bundesen et al., 2003). In an SCI compression injury model, fibrotic scar fibroblasts transiently increased Wnt/β-catenin signaling (Yamagami et al., 2018), and since Wnt signaling is known to drive fibrosis in other organs (Chilosi et al., 2003), and Wnt ligand expression is increased following SCI (Fernández-Martos et al., 2011; González-Fernández et al., 2014), induction of fibroblast WNT signaling likely contributes to fibrosis. In the photothrombotic stroke injury model, activation of TGF-β1 and retinoic acid signaling pathways in meningeal fibroblasts stimulated arachnoid barrier cells and facilitated reconstruction of the blood-CSF barrier (Cha et al., 2014). Fibroblast production of retinoic acid is also implicated as an important regulator of the post CNS injury response by several other studies. For example, perivascular fibroblasts express retinoic acid synthesizing enzymes RALDH1 and RALDH2 in uninjured brain, and the number of RALDH-expressing fibroblasts increase in the fibrotic scar following stroke injury in mice (Kelly et al., 2016). Retinoic acid signaling was elevated in neurons and astrocytes in the peri-infarct region, suggesting that scar fibroblasts signal to the surrounding CNS cells via retinoic acid release (Kelly et al., 2016). Interestingly, retinoic acid treatment following stroke in rodents reduces neurogenesis while also reducing angiogenesis and gliogenesis in the peri-infarct region (Jung et al., 2007). Thus, fibroblast production of retinoic acid seems to impair CNS recovery while stimulating fibrosis. Overall, continued investigations that detail the cellular and molecular behaviors of post injury CNS fibroblasts will forward our understanding of damage induced pathology and provide therapeutic insight.

### Meningeal Immune Cells in Acute CNS Injury

The degree of neuroinflammation following acute CNS injury is a major predictor of clinical outcomes, and numerous studies show that meningeal immune cells play an important role in the brain injury response (reviewed in Arac et al., 2014; Corps et al., 2015; Iadecola et al., 2020). In a study investigating the contribution of meninges-located mast cells in stroke pathology, depletion of mast cells resulted in decreased levels of neuroinflammation, brain swelling, and infarct size following stroke and this was partially mediated by mast-cell derived IL-6 (Arac et al., 2019; Figure 3C). Similarly, in a sub-arachnoid hemorrhage model, depletion of macrophages in the leptomeninges and perivascular spaces surrounding large arterioles prior to injury results in improved neurological scoring and reduced levels of inflammation and neuronal cell death (Wan et al., 2021).

Resident meningeal macrophages regulate the acute injury response by influencing the severity of neuroinflammation and fibrosis (Figure 3C). For example, in a mild closed-skull compression injury model, Cx3cr1+ meningeal macrophages are observed dying within 30 min of injury induction, leading to formation of a “honeycomb” network of microglia at the glial limitans, likely preventing the spread of reactive oxygen species from the leptomeninges into the brain parenchyma (Roth et al., 2013). In both MCAO and SCI models, macrophages are observed in the fibrotic scar and may associate with fibroblasts (Zhu et al., 2015; Kelly et al., 2016). One study showed that CD11b+ macrophages are partially responsible for recruitment of fibroblasts to the fibrotic scar following SCI (Zhu et al., 2015). The molecular mechanisms by which macrophages in the injury lesion could activate fibroblasts is unclear, though depletion of macrophages prior to SCI leads to depletion of pro-fibrotic cytokines, such as tumor necrosis factor superfamily members Tnfsf8 and Tnfsf13 (Zhu et al., 2015). Inhibition of pro-fibrotic cytokines could serve as a therapeutic to reduce fibrotic scarring and improve axon regrowth.

### Meningeal Lymphatics in Acute CNS Injury

Several recent studies have highlighted the contributions of dual meningeal lymphatics, and more recently, brain perivascular glymphatics, in the neuroinflammatory response following acute CNS injury (Iliff et al., 2014; Plog et al., 2015; Rasmussen et al., 2018). The brain glymphatic and meningeal lymphatic systems work together to clear waste and macromolecules from the brain parenchyma in homeostasis and following injury (Iliff et al., 2014; Plog et al., 2015; Rasmussen et al., 2018). Following TBI, glymphatic fluid flow is disrupted and greater amounts of tau protein is observed accumulating in the parenchyma (Iliff et al., 2014). Additionally, knock-out of *Aqp4*, a water transport channel necessary for interstitial solute clearance and glymphatic function, following TBI, exacerbates the accumulation of tau in the parenchyma (Iliff et al., 2014). Consistent with these results, disruption of glymphatic function results in lower levels of TBI-associated biomarkers in the cervical lymph nodes and bloodstream (Plog et al., 2015). These results highlight the important function of the glymphatic system in clearance of molecular substances from the brain and potential role in facilitating signaling between the brain and periphery.

The dural lymphatic function is also disrupted following acute CNS injury. In a mild closed-skull TBI model, meningeal lymphatic drainage is impaired likely due to increases in intracranial pressure, accompanied by lymphatic morphological changes (Bolte et al., 2020). Interestingly, lymphangiogenesis was observed up to 2 weeks following TBI, though this did not correspond with increased lymphatic function (Bolte et al., 2020). Similarly, lymphangiogenesis was seen following photothrombotic stroke lesion in the mouse cortex, but this effect was not seen following MCAO (Yanev et al., 2020; Figure 3D). Thus, the degree of lymphatic impairment and response is dependent on the injury mechanism, and there is likely communication of lymphatic vessels with surrounding tissues following injury.

The importance of meningeal lymphatics post-injury is further highlighted by studies that selective ablation of meningeal lymphatic vessels worsens pathophysiology (Figure 3D). In a subarachnoid hemorrhage model, erythrocytes accrue in the cervical lymph nodes via meningeal lymphatics following injury, and ablation of meningeal lymphatic vessels leads to less erythrocyte aggregation and greater neuroinflammation and neurological defects (Chen et al., 2020). Consistent with this observation, pre-existing defects in meningeal lymphatics causes worsened neuroinflammation following TBI (Bolte et al., 2020). Likewise, stimulating meningeal lymphatic vessel outgrowth via VEGF-C administration had decreased levels of gliosis (Bolte et al., 2020). Manipulating VEGF-C/VEGFR3 signaling is a promising avenue for mitigating post-injury complications related to meningeal lymphatic drainage. Blockage of VEGFR3 (expressed by lymphatic endothelial cells) in mice following focal cerebral ischemia resulted in reduced inflammatory response and infarct (Esposito et al., 2019). However, VEGFR3 mutant mice subjected to MCAO develop larger stroke volumes, thus worsened edema (Yanev et al., 2020). Altogether, meningeal lymphatics play a key role in regulating the post-injury response, and further work is needed to tease apart the context-dependent functions and key signaling mechanisms involved in meningeal lymphatic response to acute CNS injury.

### Response of the Meninges to Meningitis

A number of infectious agents can induce meningitis, or inflammation of the meninges, including viruses, bacteria, parasites, or fungus (McGill et al., 2017; Wright et al., 2019). Meningitis induces a variety of acute complications (severe headache, fever, photophobia, and neck pain) and regularly induces a range of long term neurological sequelae and sleep defects (Schmidt et al., 2006; Lucas et al., 2016; McGill et al., 2018). Meningitis induced inflammation is driven by the response of resident meningeal immune cells, meningeal fibroblasts and infiltrating leukocytes to the infectious agent (Coles et al., 2017; Manglani and McGavern, 2018; Rua and McGavern, 2018). For example, during murine CNS infections, including mouse hepatitis virus, a murine coronavirus (strains Srr7, cl-2, and A59), the Armstrong strain of lymphocytic choriomeningitis virus (LCMV), all show there is an influx into the brain and subarachnoid space of CD8^+^ T cells, monocytes and neutrophils into the meninges quickly after infection (Kim et al., 2009; Takatsuki et al., 2009; Cupovic et al., 2016; Watanabe et al., 2016). However, the specific localization of immune cells and neuropathology vary by strain, administration, and dose. Interestingly, in the mouse model of corona virus MHV A59 infection, production of cytokines CCL19 and CCL21 by a meningeal fibroblasts subpopulation, marked by ER-TR7 antigen, and podoplanin, work to recruit anti-viral CD8+ T cells (Cupovic et al., 2016; Figure 3B). The role of meningeal fibroblasts in fighting viral infection shares similarities to the function of reticular fibroblasts in the lymph nodes and spleen that help drive immune responses by creating specialized microenvironments (Cupovic et al., 2016; Perez−Shibayama et al., 2019; Morgado et al., 2020). In the LCMV Armstrong mouse model, it was shown that the recruitment of antiviral CD8^+^ T cells, while necessary to defeat the infection, can also be deleterious. These CD8^+^ T cells also produce cytokines that can lead to over infiltration of monocytes and neutrophils that drive breakdown of vascular integrity and induce severe edema and brainstem herniation (Kim et al., 2009; Mastorakos and McGavern, 2019), which can ultimately lead to coma and death due to the complex interplay of vascular, hypoxic, and inflammatory changes.

In cases of bacterial meningitis, infections usually start as skin, gastrointestinal or respiratory infections that spread to the blood (Doran et al., 2016; Cain et al., 2019). Once in the blood, the meningitis causing bacteria accumulate in the leptomeningeal vasculature (Mook-Kanamori et al., 2011; Iovino et al., 2013) and move into the meninges by inducing BBB breakdown through multiple mechanisms (van Sorge and Doran, 2012; Kim et al., 2015; Coureuil et al., 2017). Bacterial presence in the meninges induces a strong inflammatory response, including production of multiple cytokines (tumor necrosis factor (TNF)-alpha, interleukin (IL)-1-beta, and IL-6), and a massive infiltration of neutrophils (Mook-Kanamori et al., 2011; Principi and Esposito, 2020). The inflammatory cytokines and neutrophils work to quell the infection but also drive the breakdown of endothelial cell tight junctions (Banerjee et al., 2011; Barichello et al., 2011; van Sorge and Doran, 2012; Figure 3C).

While the reaction of meningeal immune and blood vessels to bacterial meningitis infection has been fairly well characterized, the response of meningeal fibroblasts and arachnoid barrier cells has only just begun to be investigated. All major meningitis causing bacteria can adhere strongly to meningeal fibroblasts and arachnoid barrier-like cells (Hardy et al., 2000; Fowler et al., 2004; Alkuwaity et al., 2012; Auger et al., 2015). The Doran laboratory has shown that Group B Streptococcus bacteria cross the blood brain barrier by binding to vimentin on endothelial cells (Deng et al., 2019). Vimentin is also highly expressed by arachnoid barrier cells (Weller et al., 2018), raising the possibility that this is an additional mechanism of entry for meningitis-causing bacteria, however this has yet to be experimentally assessed. Local cytokine production by meningeal immune cells may also disrupt arachnoid barrier integrity; intracisternal injection of IL1-β, highly upregulated in meningitis, induces rapid meningeal barrier leakage (Ichikawa and Itoh, 2011).

Overall, the pathology of meningitis is driven by a complex interplay between cells of the meninges and the peripheral immune system. Future work to detail the pathological mechanisms will lead to more effective treatments, improved initial diagnostics, and an enhanced understanding of what drives long term sequalae.

### Meninges and Perivascular Fibroblasts in Multiple Sclerosis

The leptomeninges are now recognized as a key player in multiple sclerosis (MS), an inflammatory CNS autoimmune disorder characterized by axon demyelination (Russi and Brown, 2015; Pikor et al., 2017; Rua and McGavern, 2018; Wicken et al., 2018). In both MS patients and MS mouse models, immune cells infiltrate the leptomeninges and pathology is commonly seen in cortical areas adjacent to the meninges (Lucchinetti et al., 2011; Mitsdoerffer and Peters, 2016; Pikor et al., 2017). In the experimental autoimmune encephalomyelitis (EAE) mouse model of MS, autoreactive effector T cells first infiltrate the leptomeninges via the pial vasculature, are activated by antigen presenting meningeal/perivascular macrophages, and subsequently enter the CNS parenchyma, triggering lesion formation (Bartholomäus et al., 2009; Schläger et al., 2016). Biopsies taken from early MS patients with cortical demyelination were more likely to have leptomeningeal inflammation, consisting of effector T cells and IgA B cells infiltrates, than those without cortical demyelination. Furthermore, in human cases of MS the severity of cortical lesions is correlated to the extent of meningeal inflammation (Lucchinetti et al., 2011). The presentation of MS induced leptomeningeal inflammation can be variable, ranging from disorganized collections of immune cells to organized ectopic lymphoid follicle-like structures (Choi et al., 2012; Mitsdoerffer and Peters, 2016; Wicken et al., 2018). Emerging work suggests that the ectopic lymph structures seen in MS are potentiated by leptomeningeal fibroblasts which form a reticular cell network consisting of activated fibroblasts in a scaffold-like structure, and that these fibroblasts “scaffolds” might be driven by a subset of meningeal fibroblast that express podoplanin (Pikor et al., 2015, 2017). This is consistent with another report showing that lymphoid follicle-like structures in the cerebral leptomeninges contained CD20^+^ B-cells, CD8^+^, CD4^+^, and CD3^+^ T-cells, CD138^+^ plasma cells, and a network of CD21^+^ and CD35^+^ follicular dendritic cells (Serafini et al., 2004). It remains unclear if ectopic lymph structures in the leptomeninges potentiate MS pathology or forms because of the pathology, but the meninges are clearly an important player in MS induced neuroinflammation.

Like acute CNS injury, CNS fibroblast activation and expansion contribute to MS induced lesions. Using a *Col1a1-GFP* mouse line in conjunction with an EAE model, two independent groups identified substantial increases in fibroblasts within spinal cord lesion sites (Yahn et al., 2020; Dorrier et al., 2021). Fibroblast expansion was deleterious for oligodendrocyte precursor cell (OPC) differentiation into mature oligodendrocytes and OPC migration into the lesion, suggesting that fibrosis may be deleterious for remyelination (Yahn et al., 2020). Of interest, a scRNAseq analysis showed that fibroblasts from EAE lesions upregulate interferon γ (IFNγ) signaling, and conditional deletion of IFN receptor γ from fibroblasts partially blocked fibroblast expansion (Dorrier et al., 2021). IFNγ is produced by spinal cord T cells in EAE mice, implicating immune cells in EAE lesions as a source of fibroblast activation signals (Dorrier et al., 2021; Figure 3C). Increased number of PDGFRβ-expressing cells are detected in human MS pathology samples (Dias et al., 2020) and increased ECM protein deposition is a common pathological feature of MS lesions (van Horssen et al., 2006), further supporting fibroblast driven fibrosis as a feature of MS. Continued studies on the mechanisms and consequences of MS induced fibrosis will likely provide therapeutic insight.

BBB breakdown is a well-documented feature of MS (Russi and Brown, 2015; Russi et al., 2018; Sweeney et al., 2019) and there is some evidence that MS can also induce disruption of the arachnoid barrier in the meninges, part of the B-CSF barrier (Bartholomäus et al., 2009; Schläger et al., 2016; Uchida et al., 2019b). Claudin 11 is a tight junction protein enriched in arachnoid barrier cells and it is downregulated in late stages of an EAE mouse model (Uchida et al., 2019b). The mechanism of downregulation and if this translates into functional breakdown is currently unknown, however, it may relate to cytokines produced by local immune cell infiltration into the meninges. Previous studies have indicated that cytokine administration into the cisterna magna of mice is sufficient to drive arachnoid barrier and blood-cerebral spinal fluid barrier functional breakdown, however this has not been shown definitively in the EAE models or MS patients (Ichikawa and Itoh, 2011). Activated T cells in the leptomeninges produce cytokines known to perturb tight junction integrity of brain endothelial cells, including IFNγ which is known to drive BBB breakdown in viral encephalitis (Bonney et al., 2019; Figure 3C). A disruption to the arachnoid barrier could permit dural and peripheral immune cells to migrate into the leptomeninges, and potentially contribute to leptomeningeal immune infiltrate that is common in MS pathology. However, more detailed investigations into the impact and cellular signals that drive this infiltration still need to be conducted. Overall, the meninges play a critical role in MS and continued work to uncover its exact contributions hold high clinical relevance.

### The Meninges and Alzheimer’s Disease

Several lines of evidence implicate meningeal-located structures in the genesis and progression of Alzheimer’s disease (AD) (for recent reviews see Da Mesquita et al., 2018a; Rasmussen et al., 2018; Nedergaard and Goldman, 2020). AD is a dementia inducing neurodegenerative disorder characterized by the accumulation of amyloid-β containing plaques, which were initially isolated from homogenates of AD patient meningeal tissue (Joachim et al., 1988; Da Mesquita et al., 2018b). The transport of interstitial fluids and macromolecules (including amyloid-β peptides) out of the brain occurs via a complex transport network that utilizes the perivascular glymphatic system of the brain and lymphatic vessels of the meninges (Rasmussen et al., 2018; Nedergaard and Goldman, 2020). Multiple publications show that both the brain glymphatic and meningeal lymphatic systems deteriorate with age (Kress et al., 2014; Ma et al., 2017; Da Mesquita et al., 2018b; Ahn et al., 2019) and their dysfunction can potentiate AD pathology and dementia (Peng et al., 2016; Da Mesquita et al., 2018a,b; Rasmussen et al., 2018; Wang L. et al., 2019; Nedergaard and Goldman, 2020; Figure 3D). For example, disruption of meningeal lymphatic vessels promotes amyloid-β deposition, in both the brain and meninges (Da Mesquita et al., 2018b; Wang L. et al., 2019), while the rescue of age-induced meningeal lymphatic defects improved cognitive performance (Da Mesquita et al., 2018b). In the APP/PS1 mouse model of AD, glymphatic dysfunction as measured by CSF clearance rates was impaired (Peng et al., 2016), and injection of amyloid-β into the CSF reduced glymphatic activity (Wang L. et al., 2019). Further, the ablation of meningeal lymphatics impedes anti-amyloid-β therapy by exacerbating microgliosis, neurovascular dysfunction, and behavior defects in the 5XFAD model of Alzheimer’s disease (Da Mesquita et al., 2021). Together this supports that impairment of meningeal lymphatics is a feature of AD and a potential therapeutic target.

Meningeal blood vessels, fibroblasts and macrophages are also of interest in AD pathology but their exact roles are unclear. There is overwhelming evidence for cerebrovascular alterations in AD pathology and amyloid-β deposition around leptomeningeal vessels is a hall mark of cerebral amyloid angiopathy, occurring in almost all AD patients (Greenberg et al., 2020). Of interest, recent scRNAseq profiling of human brain vasculature from healthy and AD cerebral cortex showed that perivascular fibroblasts were significantly under-represented in the AD brain single cell data set (Yang et al., 2021; Figure 3B). This is in contrast to large increases in CNS fibroblast numbers after acute CNS injuries and in neuroinflammation, demonstrating a different response of perivascular fibroblasts in AD. How accumulation of amyloid-β in the meningeal vasculature may potentiate neuronal impairment or impact meningeal fibroblast and immune cell populations is still being worked out. There are evidence that perivascular and barrier macrophages, found both in the brain parenchyma and leptomeninges, play a role in amyloid-β removal as their depletion causes increased amyloid deposition (Hawkes and McLaurin, 2009). Future studies that further probe AD induced changes to meningeal/perivascular fibroblasts, blood vessels and immune cells will forward our understanding of AD pathophysiology.

### Meninges as a Site of Cancer Metastasis

Primary tumors of the meninges are quite rare, however, the leptomeninges is a relatively common site for by contiguous extension of primary tumors of the central nervous system, paranasal sinuses and skull base origin or tumor metastasis which can lead to dissemination into the CNS parenchyma and poor prognosis (Mahendru and Chong, 2009; Waki et al., 2009; Oechsle et al., 2010; Scott and Kesari, 2013). Cancer cells may enter the meninges via the choroid plexus, the brain, by crossing pial blood vessels or by vascular channels that connect the bone marrow and meninges (Redmer, 2018; Yao et al., 2018). To cross the BBB, tumor cells bind endothelial cells and disrupt their tight junctions (Bos et al., 2009; Kienast et al., 2010; Fazakas et al., 2011; Redmer, 2018). Melanoma cells adhere to and disturb the interaction of brain endothelial cells, which maintain the integrity of the BBB, through a disruption of tight and adherence junction proteins such as Claudin 5 and ZO-1. In addition, proteolytic enzymes such as heparanase and seprase are important for the capacity of metastatic cells to traverse the BBB and occupy the brain (Fazakas et al., 2011). Here, micrometastases give rise to macrometastases through proliferation along brain microvessels (Kienast et al., 2010). Additionally, breast cancer cells express ST6GALNAC5, which is normally exclusively expressed in the brain, allowing for increased adhesion to brain endothelial cells to pass through the BBB (Bos et al., 2009). Further, acute lymphoblastic leukemia cells access the CNS via vascular channels that exist between bone marrow located in the vertebral and calvarium bone and the meninges (Yao et al., 2018).

Once in the leptomeninges, cancer cells can modulate the CSF content in the subarachnoid space content to support cancer growth. CSF is acellular, poor in protein, glucose and cytokine content, which is not conducive to cancer cell proliferation, however, cancer cells are able to grow in the nutrient deficient CSF filled leptomeninges using several notable mechanisms. For example, tumor cells present in the CSF secrete complement component 3 (Boire et al., 2017), which disrupts the barrier functions of choroid plexus epithelial cells, allowing nutrients and macromolecules to enter the CSF (Boire et al., 2017). scRNAseq analysis done on tumor cells isolated from the leptomeninges of patients with metastatic growth showed high expression of the iron-binding protein lipocalin-2 (Lcn2) its receptor *SLC22A17* (Chi et al., 2020). Lcn2 expression, which is induced by macrophage cytokine release, allows tumor cells to grow more effectively in the low nutrient CSF environment. Thus, tumor cells can effectively alter the leptomeninges microenvironment, however, how tumor cells effect leptomeningeal fibroblasts has not been well-studied. Continued studies to the detail the interactions between cancer cells and the leptomeninges could help develop new treatments.

## Conclusion

Here we have sought to summarize many decades of research on the cellular composition and structures of the meninges, as well as their development and function in the health CNS and contribution to injury and disease. Recent technical advances such as 2-photon *in vivo* imaging in rodents, transgenic mouse lines to better visualize meningeal cell subtypes, scRNAseq to appreciate meningeal cell heterogeneity as well as key conceptual advances in meningeal function (ex: meningeal lymphatics, connection to glymphatics, meningeal immune cell function) has enabled important new discoveries about how the meninges serves as a key interface between the CNS and periphery. There are several novel areas of meningeal biology, particularly as it relates to meningeal fibroblasts and arachnoid barrier, that we would like to highlight as areas of research in the future as well as current challenges that need to be overcome (Table 1). Further examination into the precise functions of meningeal subpopulations during homeostasis and disease may provide important insights to develop novel treatments for CNS disorders. Improved capacity to target exact subpopulations of meningeal cells may allow us to slow or halt pathogenesis and restore CNS health by reducing meningeal inflammation and barrier breakdown.

**TABLE 1:** Future areas of investigation in meninges biology.

Topic	Future areas of investigation
Regionalization	Meningeal fibroblasts show regional gene expression during development, does embryonic regionalization persist in the adult?
	Do meningeal fibroblasts have CNS region-specific functions in the healthy CNS or during disease and injury?
Layer-specific meningeal functions	Layer-specific meningeal stroma/fibroblast populations can impact specific subpopulations of immune cells (Rustenhoven, 2021), do meningeal fibroblasts in other layers serve a similar function(s)?
	Are fibroblasts spatially heterogenous (perivascular vs. layer fibroblasts) in their functions that influence immune and vascular populations?
Arachnoid barrier	What controls the development of the epithelial-like arachnoid barrier vs. arachnoid fibroblasts cells?
	What are the range of functions for the arachnoid barrier and are these functions different between development and adulthood?
	Is a “leaky” arachnoid barrier related to acute or chronic CNS insults?
Dura vasculature	When and how does the dural blood vasculature develop? What mechanisms regulate development and maintenance of diverse vascular properties in the fenestrated blood dura (as opposed to the barrier leptomeninges vasculature)?
CNS fibroblast identity	Many whole brain single cell studies annotate fibroblast containing clusters as “vascular leptomeningeal cells,” while other studies refer to these cells as stromal cells, mesenchymal cells, Type A Pericytes, Type 2 pericytes and fibroblasts. There is a lack of consensus on the spatial, transcriptional, and potential functional heterogeneity for these populations. Consistent annotation and analysis are needed to fully advance studies of different CNS fibroblast populations.

*This table elucidates the prioritized areas of investigation on key topics that need further study, including: meningeal cellular regionalization, layer-specific functions, arachnoid barrier development and function, dural vasculature development, and CNS fibroblast identity.*

## Author Contributions

JD, HEJ, CC, BP, and JS contributed to conceptualization, writing, and editing of the review. All authors contributed to the article and approved the submitted version.

## Conflict of Interest

The authors declare that the research was conducted in the absence of any commercial or financial relationships that could be construed as a potential conflict of interest.
