# Corrigendum: Living on the Edge of the CNS: Meninges Cell Diversity in Health and Disease

**DOI:** 10.3389/fncel.2021.761506

**Published:** 2021-10-08

**Authors:** Julia Derk, Hannah E. Jones, Christina Como, Bradley Pawlikowski, Julie A. Siegenthaler

**Affiliations:** ^1^Section of Developmental Biology, Department of Pediatrics, University of Colorado, Aurora, CO, United States; ^2^Cell Biology, Stem Cells and Development Graduate Program, University of Colorado, Anschutz Medical Campus, Aurora, CO, United States; ^3^Neuroscience Graduate Program, University of Colorado, Aurora, CO, United States

**Keywords:** meninges, fibroblast, meningeal lymphatic system, arachnoid barrier, blood-CSF barrier, border-associated macrophages

In the original article **Yao et al.**, **2018** was not cited in the article. The citation has now been inserted in ***Meningeal cell types, Immune cells, Paragraph 3*** and should read:

Macrophages of the meninges are one of better documented meningeal immune cell and belong to a highly specialized class of macrophages called border associated macrophages (BAMs). BAMs and microglia both originate from yolk sac erythro-myeloid progenitors and can be detected in the brain as early as E10 (Utz et al., 2020). As development continues BAMs and microglia segregate both physically and transcriptionally, with BAMs remaining in the leptomeninges (they are also in the choroid plexus and perivascular spaces) and expressing *CD206* and *Lyve1* which are not expressed by microglia. In the adult, leptomeningeal BAMs are defined by expression of *CD206, Lyve1, P2rx7*, and *Egfl7* and have significantly different transcriptional profiles from dural BAMs (Mrdjen et al., 2018; Van Hove et al., 2019). Adult dural BAMs don't express *Lyve1* and can also be divided in subgroups. For example, one group of dural BAMs has low expression of major histocompatibility complex II (MHCII^lo^) and express *Clec4n, Clec10a, Folr2*, while MHCII^hi^ dural BAMs express greater CCR2, implicating a monocytic origin. Another important difference is that leptomeningeal BAMS are long lived while dural BAMs are continuously renewed by peripheral monocytes (Goldmann et al., 2016; Van Hove et al., 2019). The bone marrow in the calvarium and vertebral column specifically supply monocytes and neutrophils to the dura during homeostasis (Cugurra et al., 2021) and to the meninges and brain parenchyma following brain injury or in neuroinflammation via vascular tunnels connecting the bone marrow and dura (Herisson et al., 2018; Yao et al., 2018; Cai et al., 2019; Cugurra et al., 2021). The unique properties seen among leptomeningeal and dural BAMs is consistent with specialized functions for these populations in their respective barrier and non-barrier compartments.

In the original article, there was an error. We omitted reference to an important access mechanism for cancer cells to enter the CNS, vascular channels from the calvarial bone to the meninges described in Yao et al., 2018 *Nature*.

A correction has been made to ***Meningeal response to injury and disease, Meninges as a site of cancer metastasis, Paragraph 1***:

Primary tumors of the meninges are quite rare, however, the leptomeninges is a relatively common site for by contiguous extension of primary tumors of the central nervous system, paranasal sinuses and skull base origin or tumor metastasis which can lead to dissemination into the CNS parenchyma and poor prognosis (Mahendru and Chong, 2009; Waki et al., 2009; Oechsle et al., 2010; Scott and Kesari, 2013). Cancer cells may enter the meninges via the choroid plexus, the brain, by crossing pial blood vessels or by vascular channels that connect the bone marrow and meninges (Redmer, 2018; Yao et al., 2018). To cross the BBB, tumor cells bind endothelial cells and disrupt their tight junctions (Bos et al., 2009; Kienast et al., 2010; Fazakas et al., 2011; Redmer, 2018). Melanoma cells adhere to and disturb the interaction of brain endothelial cells, which maintain the integrity of the BBB, through a disruption of tight and adherence junction proteins such as Claudin 5 and ZO-1. In addition, proteolytic enzymes such as heparanase and seprase are important for the capacity of metastatic cells to traverse the BBB and occupy the brain (Fazakas et al., 2011). Here, micrometastases give rise to macrometastases through proliferation along brain microvessels (Kienast et al., 2010). Additionally, breast cancer cells express ST6GALNAC5, which is normally exclusively expressed in the brain, allowing for increased adhesion to brain endothelial cells to pass through the BBB (Bos et al., 2009). Further, acute lymphoblastic leukemia cells access the CNS via vascular channels that exist between bone marrow located in the vertebral and calvarium bone and the meninges (Yao et al., 2018).

The authors apologize for this error and state that this does not change the scientific conclusions of the article in any way. The original article has been updated.

## Publisher's Note

All claims expressed in this article are solely those of the authors and do not necessarily represent those of their affiliated organizations, or those of the publisher, the editors and the reviewers. Any product that may be evaluated in this article, or claim that may be made by its manufacturer, is not guaranteed or endorsed by the publisher.
